# Skin models of cutaneous toxicity, transdermal transport and wound repair

**DOI:** 10.1093/burnst/tkad014

**Published:** 2023-07-28

**Authors:** Inês Vilela de Sousa, Miguel J S Ferreira, Luís B Bebiano, Sandra Simões, Ana Filipa Matos, Rúben F Pereira, Pedro L Granja

**Affiliations:** i3S - Instituto de Investigação e Inovação em Saúde, Universidade do Porto, Rua Dr. Manuel Pereira da Silva, 4200-393 Porto, Portugal; INEB - Instituto de Engenharia Biomédica, Universidade do Porto, Rua do Campo Alegre, 823, 4150-180 Porto, Portugal; ICBAS – Instituto de Ciências Biomédicas Abel Salazar, Universidade do Porto, Rua de Jorge Viterbo Ferreira 228, 4050-313 Porto, Portugal; FEUP – Faculdade de Engenharia da Universidade do Porto, Rua Dr. Roberto Frias, 4200-465 Porto, Portugal; i3S - Instituto de Investigação e Inovação em Saúde, Universidade do Porto, Rua Dr. Manuel Pereira da Silva, 4200-393 Porto, Portugal; INEB - Instituto de Engenharia Biomédica, Universidade do Porto, Rua do Campo Alegre, 823, 4150-180 Porto, Portugal; ICBAS – Instituto de Ciências Biomédicas Abel Salazar, Universidade do Porto, Rua de Jorge Viterbo Ferreira 228, 4050-313 Porto, Portugal; FEUP – Faculdade de Engenharia da Universidade do Porto, Rua Dr. Roberto Frias, 4200-465 Porto, Portugal; Department of Mechanical, Aerospace and Civil Engineering, School of Engineering, Faculty of Science and Engineering & Henry Royce Institute, Engineering Building A, The University of Manchester, Oxford Road, Manchester M13 9PL, UK; i3S - Instituto de Investigação e Inovação em Saúde, Universidade do Porto, Rua Dr. Manuel Pereira da Silva, 4200-393 Porto, Portugal; INEB - Instituto de Engenharia Biomédica, Universidade do Porto, Rua do Campo Alegre, 823, 4150-180 Porto, Portugal; ISEP - Instituto Superior de Engenharia do Porto, Universidade do Porto, Rua Dr. António Bernardino de Almeida 431, 4200-072 Porto, Portugal; iMed.ULisboa, Faculty of Pharmacy, Universidade de Lisboa, Av. Prof. Gama Pinto, 1649-003 Lisboa, Portugal; Faculty of Pharmacy, Universidade de Lisboa, Av. Prof. Gama Pinto, 1649-003 Lisboa, Portugal; i3S - Instituto de Investigação e Inovação em Saúde, Universidade do Porto, Rua Dr. Manuel Pereira da Silva, 4200-393 Porto, Portugal; INEB - Instituto de Engenharia Biomédica, Universidade do Porto, Rua do Campo Alegre, 823, 4150-180 Porto, Portugal; ICBAS – Instituto de Ciências Biomédicas Abel Salazar, Universidade do Porto, Rua de Jorge Viterbo Ferreira 228, 4050-313 Porto, Portugal; i3S - Instituto de Investigação e Inovação em Saúde, Universidade do Porto, Rua Dr. Manuel Pereira da Silva, 4200-393 Porto, Portugal; INEB - Instituto de Engenharia Biomédica, Universidade do Porto, Rua do Campo Alegre, 823, 4150-180 Porto, Portugal; ICBAS – Instituto de Ciências Biomédicas Abel Salazar, Universidade do Porto, Rua de Jorge Viterbo Ferreira 228, 4050-313 Porto, Portugal; FEUP – Faculdade de Engenharia da Universidade do Porto, Rua Dr. Roberto Frias, 4200-465 Porto, Portugal

**Keywords:** Skin, *In vitro* models, *Ex vivo* models, Transdermal transport assays, Wound healing assays, Toxicity assays, Microfluidic chip, Skin-on-a-chip

## Abstract

Skin is widely used as a drug delivery route due to its easy access and the possibility of using relatively painless methods for the administration of bioactive molecules. However, the barrier properties of the skin, along with its multilayer structure, impose severe restrictions on drug transport and bioavailability. Thus, bioengineered models aimed at emulating the skin have been developed not only for optimizing the transdermal transport of different drugs and testing the safety and toxicity of substances but also for understanding the biological processes behind skin wounds. Even though *in vivo* research is often preferred to study biological processes involving the skin, *in vitro* and *ex vivo* strategies have been gaining increasing relevance in recent years. Indeed, there is a noticeably increasing adoption of *in vitro* and *ex vivo* methods by internationally accepted guidelines. Furthermore, microfluidic organ-on-a-chip devices are nowadays emerging as valuable tools for functional and behavioural skin emulation. Challenges in miniaturization, automation and reliability still need to be addressed in order to create skin models that can predict skin behaviour in a robust, high-throughput manner, while being compliant with regulatory issues, standards and guidelines. In this review, skin models for transdermal transport, wound repair and cutaneous toxicity will be discussed with a focus on high-throughput strategies. Novel microfluidic strategies driven by advancements in microfabrication technologies will also be revised as a way to improve the efficiency of existing models, both in terms of complexity and throughput.

Highlights

- *In vitro* and *ex vivo* skin models have been emerging as alternatives to animal models, but for these new platforms to gain regulatory approval they should closely resemble *in vivo* skin behaviour, use standardized methodologies and provide reproducible and predictive results.
- A high-throughput model corresponds to a faster method that allows a greater number of samples to be processed at the same time.
- The emergence of more complex microfluidic platforms that simulate the skin in health and disease is enabling the study of multiple processes such as transdermal transport, wound healing and toxicity, thus contributing to the reduction of animal testing.

## Background

The skin functions not only as a mechanical and chemical defence system of the human body but also as a relevant immune and sensory organ [1,2]. Skin is composed of three main layers: epidermis, dermis and hypodermis [3]. Its singular properties give it a paramount barrier role, protecting the human body not only against external agents but also in promoting homeostasis [4].

The skin has also emerged as an important route for drug delivery. A myriad of treatments using the skin as the target organ for drug administration are already commercially available not only for the treatment of skin ailments but also for other medical conditions (e.g. chronic pain, tobacco cessation, contraception) [5–7]. The easy access and use of relatively painless methods of administration, in addition to circumventing the hepatic first-pass metabolism, justify the choice of skin for drug administration [7–12]. Even though skin barrier properties are crucial for homeostasis, they pose a great challenge for drug delivery, as only small molecules with high lipophilicity can cross it. Therefore, strategies to circumvent the skin barrier properties have been the focus of multiple research efforts [7,10]. However, since the skin serves as the main barrier between the body and the external environment, its disruption can lead to serious health problems, such as infection and chronic wounds, having a great social and economic impact on the lives of millions of patients [13]. Hence, the development of new strategies for improving wound repair and regeneration is also of paramount importance. Furthermore, there is a wide range of chemical substances that can have detrimental toxicological consequences for human health through skin contact, leading also to its disruption, thus requiring specific testing for their safety assessment [14].

Regardless the application is for drug delivery, toxicity assessment, or fundamental biology research, *in vitro* assays are an essential part of the process. Despite static 2D *in vitro* cell culture studies providing valuable information, they are limited in their ability to reproduce the native 3D tissue microenvironment and cell behaviour, making it necessary to develop more complex models [15–17]. Advances in 3D tissue engineering and microfluidics have enabled the development of more advanced and complex *in vitro* assays that more closely resemble physiological conditions [15,17–23]. These more advanced methods have been emerging as alternatives to *in vivo* animal studies, as they provide increased predictive power over other 2D *in vitro* assays, and thus contribute to the application of the 3Rs principle regarding animal welfare [24,25]. Nevertheless, in order for these platforms to gain regulatory approval, they should closely resemble *in vivo* skin behaviour, use standardized methodologies and provide reproducible and predictive results [26].

Herein, we provide an overview of skin models for transdermal transport, wound repair and cutaneous toxicity assessment, taking into consideration their compliance with international testing guidelines and current research needs. Moreover, advanced technologies, such as organ-on-a-chip models for skin applications, will be discussed with regard to their main features and upcoming potential for modelling skin functions and properties.

## Review

### *In vitro* and *ex vivo* assays for transdermal transport assessment

Transdermal drug delivery is based on the application of a substance directly on intact skin, leading to penetration through different skin layers prior to absorption into the systemic circulation as it reaches the dermis [27,28]. Transport through the skin can occur across the sebaceous glands or hair follicles (appendageal route), between the cells (intercellular route) or across the cells (transcellular route) [29,30].

It has long been known that the top layer of the epidermis, the stratum corneum (SC), is the most important barrier for transdermal transport [31]. The SC is composed of terminally differentiated epidermal keratinocytes and is characterized by having a ‘bricks and mortar’ structure, with keratin-rich corneocytes acting as ‘bricks’ embedded in extracellular hydrophobic ‘mortar’ [32–34]. Hence, the SC acts as an entry barrier for non-lipidic or high molecular-weight compounds [7,23,28,32]. Underneath the SC, a more hydrophilic layer, commonly named the viable epidermis, is composed of keratinocytes at different stages of differentiation, Merkel cells, Langerhans cells and melanocytes. Thus, the viable epidermis can be a rate-limiting step to transdermal penetration, making it relevant to study not only the influence of the SC but also of the remaining skin layers in transdermal transport studies [35,36]. Both the thickness of the dermis and the composition of the extracellular matrix (ECM) have a significant impact on the efficiency of transdermal transport. In addition to skin thickness, lipid content, hair follicle density and enzyme activity in each model can also act as a source of variability when predicting transdermal transport [37].

Based on the origin of the skin, transdermal transport models can be classified as *ex vivo,* when the skin is obtained from humans/animals, or *in vitro,* with skin constructs provided by human primary cell culture, forming *in vitro* human skin equivalents (HSE) [38]. There is also the possibility of using cell-free, lipid-coated membrane models as skin surrogates [39]. According to the organization for economic cooperation and development (OECD) guidelines, viable human skin obtained from autopsies or surgery, which without any other end-use would be considered biological waste, is the gold standard for transdermal transport studies [37,38,40,41]. However, not only is its availability limited but also a high variability is usually observed among donors in terms of age, gender, ethnicity and even biopsy site [38,42]. Unlike human skin, animal skin is more readily available and can be more easily extracted in a timeframe closer to use [42]. Some of the commonly used animal skin sources include rodents, rabbits and pigs [43–46]. Although porcine skin is histologically more similar to human skin, rodent skin (specifically from rats) is the most widely used animal source for transdermal transport studies. This is due to the lower cost, higher availability and easier handling methods [37,38,47]. Nonetheless, multiple studies have reported disparity in the permeability of human and animal skin from different sources [43,44,46,48–51]. As an alternative to human or animal skin, HSE can also be used to evaluate transdermal transport [44]. These can either be composed of SC and viable epidermis (reconstructed human epidermis (RHE)) or include an additional dermal layer (full-thickness) [52,53]. These types of *in vitro* models exhibit reduced variability but tend to have enhanced permeability levels when compared to *ex vivo* human or pig skin [52,54]. Moreover, as these types of constructs usually lack skin appendages, such as hair follicles, they may not be suitable for studying some transport phenomena, such as for instance follicular penetration [46].

The OECD guidelines for testing chemical compounds recommend the use of diffusion-cell assays to investigate transdermal transport. This type of set-up is composed of two compartments—donor and receptor—separated by a skin membrane with the upper layers (SC) in contact with the donor phase. The test substance is applied to the donor chamber and left for a period of time, followed by appropriate removal. Evaluation of the substance or its metabolites can be performed in the receptor fluid, even though it is also important to consider the amount remaining absorbed in each layer of the skin [40].

As a measurement of the capacity to process samples, a distinction between low- and high-throughput methods may be considered. A low-throughput model takes longer to carry out and can only be applied to a few samples. In contrast, a high-throughput model corresponds to a faster method, which allows a greater number of samples to be processed at the same time, as a result of faster work, or the possibility of processing multiple samples at once or simultaneously handling multiple aspects of the same sample.

#### Low-throughput models

**Figure 1:**
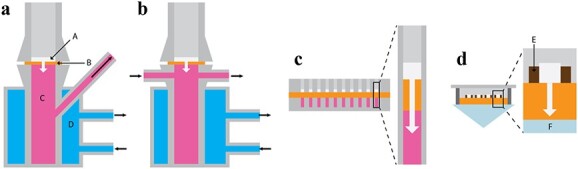
Transdermal transport models. (**a**) Franz diffusion cell; (**b**) flow-through diffusion cell; (**c**) high-throughput transdermal transport model with two plates fixed to each other; and (**d**) high-throughput transdermal transport model created with a paraffin grid. A, Test compound; B, skin sample; C, receptor fluid; D, temperature-regulating fluid; E, paraffin; F, attenuated total reflectance-Fourier transform infrared spectroscopy crystal

Franz diffusion cells (Figure 1a) are static models that were introduced in 1975 as a way to replicate percutaneous absorption and are now commercially available with widespread use [35,55–57]. Their design consists of skin samples placed over an O-ring ball joint separating two parts of a glass chamber. The epidermal side is exposed to the sample while a buffer solution is in contact with the dermal portion. The receptor medium is under magnetic agitation and controlled temperature conditions and can be removed, replaced and analysed at different time points during the transdermal transport assays [57]. Even though this model is versatile, allowing infinite and finite dose studies, it does not allow continuous perfusion of the sample and requires large skin samples [57–59].

Flow-through diffusion cells (Figure 1b) were later developed, using a perfused fluid under the skin surface flowing at a defined rate. Identically to blood flow, the receptor fluid is constantly renewed, which makes this strategy more biologically relevant to studying compounds with limited skin permeability. In addition, it allows for automatic sample collection and is more convenient for monitoring [60–62]. Pulsoni *et al.* compared the performance of a device that properly resembles the mono-directional physiological capillary-like flow below the skin to a Franz diffusion cell. They concluded that the former provides results that are more similar to those obtained *in vivo* for the permeation of a lipophilic molecule, demonstrating the importance of blood flow simulation in transdermal transport studies [56].

#### High-throughput models

The wide range of potential skin permeability enhancement strategies that can be applied alone or in combination to study transdermal delivery of different compounds makes it necessary to transition from conventional models to novel high-throughput strategies. Conventional methodologies, such as Franz diffusion cells, are not compatible with high-throughput screening since they require large skin areas and time-consuming sample set-up and manipulation [63]. Karande and Mitragotri developed a high-throughput method for up to 100 assays, allowing a 50-fold higher efficiency in skin usage when compared with a Franz diffusion cell (Figure 1c). The model is based on two plates fixed to each other and drilled with an array of 100 (10 x 10) 3 mm orifices that act as wells. The plates are separated by a skin sample, with the SC facing the donor plate and the dermal side exposed to a phosphate-buffered saline solution in the receiving plate. For validation, the skin permeability was challenged with chemical enhancers and evaluated by conductivity measurements [63]. This model has been successfully applied for screening synergic combinations of 32 different chemical enhancers in >5000 formulations [64]. Even though it has shown promising results, permeability assessment has not been automated.

Andanson *et al.* developed a different model by using a paraffin automatic dispenser that was able to draw 16 octagonal wells with a 2.4 mm diameter on a skin sample to study multiple formulations simultaneously (Figure 1d). These wells acted as impermeable barriers containing different solutions that were manually applied with a pipette. The permeation of 12 different conjugations of an enhancer and a permeant were simultaneously assessed by placing the tissue on an attenuated total reflection Fourier transform infrared (ATR-FTIR) crystal. Although this model presents limitations regarding the number of samples that can be simultaneously tested, due to the dryness and shrinkage of the edges of the skin when placed on the crystal, it also grants an important advance for the high-throughput permeation assessment of multiple samples [65].

More recently, Martins *et al*. developed a high-throughput screening system based on two attached 96-well plates, one with and the other without the bottom. The former acted as a receptor compartment, while the the latter acted as the donor compartment. Damaged skin, to increase the permeation of the model drug, was placed between the two plates, on top of the receptor compartment. The permeability of a fluorescent sulforhodamine B (hydrophilic model drug) was assessed by spectrophotometric analysis of the liquid in the receptor phase and skin retention was assessed by 2-photon microscopy. It was possible to observe similar permeation and skin retention levels between this system and Franz diffusion cells. Furthermore, statistical studies determined that this high-throughput system needs 10x fewer samples than the Franz diffusion cell to obtain statistically significant results. In addition, Martins *et al.* showed the high-throughput screening ability of this model by using a 96-well plate and testing different formulations with varying solvent ratios and different concentrations and types of permeation enhancers. They showed not only the effect of the permeant enhancer concentration on increasing skin retention in the different layers of the skin but also a possible interaction between the latter and the different solvent ratios on the top layers of the tissue. To demonstrate the possibility of higher throughput analysis, Martins *et al.* tested the permeability of two different concentrations of the drug model in a 384-well plate, obtaining higher values of permeability when higher doses of the drug were used. Nonetheless, the cross-contamination observed among the different formulations tested might hinder the use of this type of plate. Despite having developed a high-throughput model able to assess not only skin permeability but also retention, the fact that only damaged skin was tested, coupled with it being only possible to analyse fluorescent substances, limits its applicability [59].

High-throughput strategies often require miniaturization. This leads to new challenges due to changes in permeability as a consequence of the decreasing skin–compound contact area [66]. In addition, transdermal patches are widely used as drug delivery systems and represent a significant global market within transdermal medication [67]. The patches can vary in size and thickness but are themselves 3D structures designed to adhere to the skin and release the drug systemically in a prolonged and controlled way. Therefore, the evaluation of transdermal patches using high-throughput models would pose another challenge considering the tissue manipulation requirements.

Both screening strategies discussed above are viable for transdermal transportation studies. While low-throughput methods are simpler, high-throughput assays enable the testing of multiple formulations, which is fundamental in the drug-development industry. Nonetheless, there seems to be considerable room for improvement in both strategies, particularly regarding their level of throughput and automation.

### Wound healing studies

Wound healing is a complex process requiring the concerted spatiotemporal interplay of several different agents. This process is composed of four major phases: haemostasis, inflammatory, proliferative and maturation/remodelling [68,69]. The coagulation and inflammation phase is first characterized by the formation of a fibrin plug. This not only promotes haemostasis and provides temporary wound closure, but also acts as a provisional matrix for new tissue formation, as well as a reservoir of chemokines and growth factors [70,71]. During the following phase, the formation of new tissue, keratinocytes migrate, proliferate and mature over the disrupted dermis, contributing to the restoration of skin epithelial barrier function properties [68]. This process of wound re-epithelization is of great importance and its malfunction may lead to the generation of chronic wounds [72,73]. As endothelial cells form new blood vessels, some capillaries colonize the fibrin clot, giving rise, together with macrophages and fibroblasts, to granulation tissue rich in collagen type III. The presence of transforming growth factor beta (TGF-β)1, secreted not only by macrophages but also by other immune cells and dermal fibroblasts, in addition to the existing mechanical tension in the wound, stimulates fibroblast differentiation into myofibroblasts which, with their contractile activity, promote wound closure [68]. The last phase, tissue remodelling, is characterized by a decrease in cellular activity, apoptosis at the wound site and ECM remodelling, in which type III collagen is replaced with type I [68,74].

Skin wounds result in a considerable societal burden with a high economic impact. Research towards a better understanding of wound healing is of paramount importance to develop new strategies to improve wound repair and regeneration [13]. To achieve this aim, low- and high-throughput models have been developed.

#### Low-throughput models

Wound repair studies can be performed *in vitro* using 2D or 3D skin models. 2D models are focused on simple aspects of wound repair and are often easier and simpler to perform. Nonetheless, more complex 3D models provide better mimicry of tissue microenvironment, allowing the study of a wider range of phenomena [75,76]. Different assays have been developed allowing the study of chemical agents or physical stimuli, such as the influence of electric fields or shear stress [76–78].

**Figure 2:**
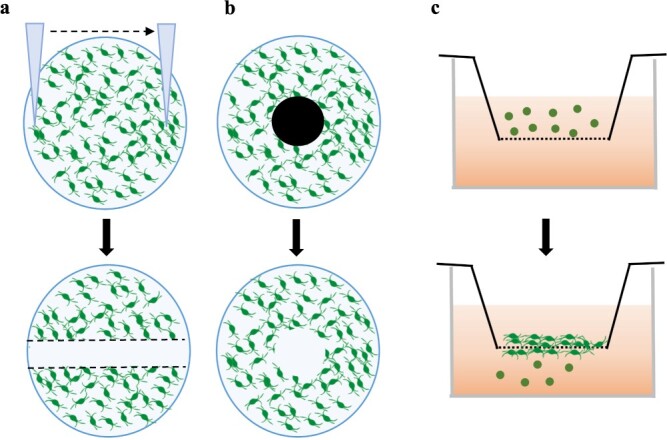
Low-throughput 2D wound healing assays. (**a**) Scratch assay; (**b**) mechanical barrier assay; (**c**) cell migration assessment using a Boyden chamber

A simple 2D model to study wound repair is the *in vitro* scratch test (Figure 2a). This strategy consists of the creation of a ‘scratch’ on a confluent cell monolayer, followed by observation of cell migration from the edges of the gap towards its centre, in a process mimicking wound closure. Despite being a very basic approach, it allows continuous monitoring of the samples with time-lapse microscopy, as well as evaluation of single-cell migration [76,79]. As an alternative to mechanical wounding, studies have been developed using barrier migration assays. In these assays, instead of disrupting a cell monolayer, a wound model is created through the use of a physical barrier insert that does not allow cell colonization of a certain area (Figure 2b) [80]. Other strategies rely on the study of cell migration using a membrane. This has been done with the Boyden chamber assay. A Boyden chamber is composed of two compartments separated by a porous membrane and loaded with a cell suspension on the top of the membrane and a test solution on the bottom (Figure 2c). After incubation, the membrane is removed from the chamber. Cell migration is then observed using microscopy techniques [81]. Modifications of this technique have been used in skin wound repair studies for evaluating the effect of both chemotactic promoters and inhibitors [82–84]. Although a very simple, quick and facile method of wound emulation, the scratch assay does not allow the creation of wounds that are exact replicas of each other, since the process is dependent on the operator. Hence, the mechanical barrier method can overcome this problem of wound reproducibility, generating less variability among the wounds due to the constant maintenance of the wounded area. Moreover, in spite of the Boyden chamber not simulating a wound as a disruption of cell lining, it offers additional information regarding cell migration in response to chemicals.

3D wound models can be created using scalpels, skin meshes, freeze damage or laser irradiation [75,85–87]. These models can better emulate wound repair, allowing a more complex study of biological processes, such as cell migration of different cell types, understanding biomarker expression, and unveiling the role of each cell activity in wound and scar formation [88]. One example is the use of scalpel incisional or excisional wounding of HSE constructs. Incisional wounds are produced by incising tissues with a scalpel, allowing the wound edges to be separated and generating an elliptical wound. In the case of excisional wounds, these can be created using an elliptical dermatological punch. The punch fully penetrates the centre of the tissue, excising the epidermis, collagen and membrane [89]. Burn wound models are another example. These aim to answer specific research questions regarding burn wound healing mechanisms [90–92]. In a general way, the burn injury is created using a pre-heated tool which is then applied to the model at different temperatures and time periods to recreate specific degrees of severity (superficial and full-thickness burns). With both assays, it is possible to observe keratinocyte migration, restoration of epithelial integrity, wound closure and stratification [75]. However, most 3D wounding strategies not only require extensive tissue manipulation but also present inherent difficulties in wound reproducibility [75,86,87]. This is mostly related to the lack of standardization regarding (1) biomaterials, cells and cell culture protocols used to establish the models, which can lead to altered cellular response and biological functionality, as well as (2) experimental methodologies used for wound generation. One way to reduce the need for manipulation while keeping reproducibility in creating wounds is to use laser irradiation. With this strategy, it is possible to control the magnitude of the damage by varying the number of laser pulses applied. This affects the epidermis alone or also the dermis, thus creating a more reliable wound model [85]. Bioprinting is also a promising strategy to fabricate 3D skin models. Commonly it involves the sequential deposition of biomaterials combined with cells (known as bioinks) to mimic either one or multiple skin layers. Generally, a hydrogel precursor solution or a pre-crosslinked hydrogel is loaded with dermal fibroblasts and bioprinted to mimic the dermal layer, followed by the bioprinting or seeding of keratinocytes to resemble the epidermis.

#### High-throughput models

These models have been developed not only for cell migration assessment on flat surfaces but also through a membrane [93,94]. Yarrow *et al.* developed a variation of the *in vitro* scratch test for high-throughput analysis in a 384-well plate. Cell monolayers were disrupted by a manually operated 96-array of pins, resulting in wounds of similar morphology in different wells. The multiple processes underlying wound healing were evaluated through different imaging techniques [94]. Recently, a similar device became commercially available [95]. An alternative strategy used the laminar flow of trypsin to generate a wound-healing model through cell detachment. By using a microfluidic model composed of poly(dimethyl siloxane) (PDMS) microchannels, scale-up towards high throughput can be more readily achieved [96]. PDMS is the most widely used material for microfluidics applications due to its adequate biological (e.g. biocompatibility) and physical–chemical (e.g. low glass transition temperature, easy processability and surface modification, gas permeability, optical transparency, soft elastomeric nature) properties associated with low cost [97]. Zordan *et al.* developed an integrated system using laser pulses to create wound model cell cultures in 96-well plates. This high-throughput system used an automated mechanism for wound generation and image capturing at different time points after wounding. Using this system, it became possible to evaluate the closure of irregularly shaped wounds without the need for sample fixation in a fast, high-throughput manner [98]. Recently, Acosta *et al.* created simple 3D-printed barrier moulds with the exact size of a 96-well plate, creating wounds with reproducible sizes. Furthermore, they not only demonstrated the maintenance of cell viability when in contact with the moulds but were also able to study the effect of different concentrations of serum on the wound healing and proliferative population of HaCaT cells [99].

High-throughput 3D models are scarce. Nonetheless, Promocell® developed a new wound healing system based on magnetic 3D bioprinting. Spheroids with different ratios of magnetically labelled fibroblasts and keratinocytes were formed in each well of a 384-well plate, using magnetic forces. Then, a magnetic 384-ring plate was placed on top, resulting in the generation of wounds with the same structure. One of the main innovations of this system is the possibility of co-culture with different cell ratios, allowing the emulation of the different phases of wound healing [100].

As previously shown, different methodologies can be applied to the observation of one single phenomenon. Advances in miniaturization, automation and image analysis are boosting the creation of systems that allow the simultaneous, quantitative evaluation of multiple reproducible wounds.

### Skin toxicity studies

Different substances and mixtures can have detrimental health consequences. Therefore, a classification and labelling system has been established by the United Nations [14]. Health hazards due to skin contact can be divided into toxicity, corrosion, irritation and sensitization. Corrosion is defined as irreversible damage, with visible necrosis in the epidermis and dermis, whereas irritation leads to reversible damage [101,102]. Sensitization occurs when there is an allergic response after contact with the skin [103]. These classification systems are mainly based on animal studies and clinical information. However, *in vitro* tests are also recommended by the OECD for the assessment of skin corrosion and irritation [101,102,104,105]. The prohibition of animal testing for cosmetic products in some regions has made mandatory the use of *in vitro* assays for toxicity, irritation, sensitization and, in some cases, photo-induced toxicity for these products [106,107]. Other studies, such as genotoxicity, may also be relevant and the OECD has issued guidelines for its *in vitro* assessment [108,109].

Skin corrosion can be evaluated *in vitro* by measurement of transepithelial electrical resistance (TEER), a parameter that evaluates SC integrity and barrier function, as recommended by OECD guidelines [104]. TEER should be evaluated after 24 h of contact with a test substance. A corrosive effect is noticeable in a decrease of TEER below a threshold level [104]. Even though this method recommends the use of rat skin, it is also possible to identify corrosive substances and mixtures using RHE models [104]. A range of RHE commercially available models, such as the EpiSkin™ [110], EpiDerm™ [111], SkinEthic™ [112], EpiCS® [113] and LabCyte EPI-MODEL [114] are recommended by the OECD [102]. Using RHE models, this method considers corrosive chemicals as those that are able to diffuse through or erode the SC, having cytotoxic effects in the underlying layers. These are evaluated by measuring cell viability immediately after chemical exposure using *in vitro* metabolic activity assays [102]. Lastly, OECD guidelines also propose the assessment of corrosion *in vitro* through the evaluation of the amount of damage inflicted by a chemical on artificial membranes [105].

All the methods discussed can only evaluate skin corrosion, and are not able to correctly distinguish skin irritants [115]. Therefore, skin irritation is evaluated with RHE models using a different protocol from corrosion [101]. In addition to the RHE commercially available models recommended for assessment of skin corrosion, two more RHE models are further validated for irritation screening [101]: Skin+® [116] and KeraSkin™ [117]. According to the OECD guidelines, the degree of skin irritation is assessed through cell viability by performing the 3-(4, 5-dimethylthiazolyl-2)-2, 5-diphenyltetrazolium bromide (MTT) test, a colourimetric assay [101].

Skin sensitization can be assessed *in vitro* with the KeratinoSens™ luciferase assay. KeratinoSens™ is an immortalized human keratinocyte cell line that expresses luciferase upon activation of a cellular stress pathway in response to exposure to a skin sensitizer. The upregulation of the luciferase gene can then be quantified by luminescence detection [103]. This assay is suitable for high-throughput screening. Nonetheless, skin sensitization can only be thoroughly evaluated in combination with other testing methodologies. Some of the suggested techniques are comparisons with chemical analogues, the study of chemical reactivity with peptides or proteins, dendritic cell activation studies in *in silico* models, and the use of RHE models [103,118–121].

Cutaneous toxicity is a complex process. Even though there have been significant improvements in *in vitro* methodologies for its evaluation, only a few protocols have proven to be robust enough to be recommended in international guidelines. Moreover, these methods alone are often not enough to gain a complete understanding of skin toxicity. Therefore, it is necessary to develop novel, integrative testing platforms for the assessment of skin toxicity. These need to be reliable, versatile and reproducible, i.e. able to be implemented in multiple laboratories.

### Microfluidic strategies for skin emulation

Due to the current need for the development of more biologically relevant platforms for *in vitro* studies, organ-on-a-chip devices have been developed. These aim to model the physiological or pathophysiological functions of tissues or organs through the use of cell culture in continuously perfused microfluidic systems [16]. Microfluidic devices for emulation of organs, such as the lung [122–128], kidney [129–133], liver [134–137], bone marrow [138–140], gut [137,141–145] and skin [146,147] have been the focus of a myriad of research groups in the past decade. These are versatile models that can be based on *in vitro* or *ex vivo* culture systems [122,138].

In recent years, there have been significant developments leading to the establishment of a range of skin-on-a-chip platforms, as summarized in Table 1. These are based on a wide variety of tissue models, from keratinocyte [148–152] or fibroblast [77,153–155] cultures to HSEs [146,147,156] or *ex vivo* tissue [157–161]. They have been applied to study transdermal transport [146,157,162–164] and wound healing [77,151,155] or to assess the response to shear stress [77,147,152–154], chemical agents [146,148–150,154,160,161,165–167] or electrical stimuli [77,151]. Furthermore, new microfluidic systems for skin disease emulation have also emerged [148,158,168,169].

**Table 1:** Types of on-chip assays performed using skin microfluidic devices

**Stimulus**	**Skin model**	**On-chip purpose**	**Reference**
Shear stress, EGF	mFb	Control cell adhesion	[154]
Shear stress	mFb	Assess effect of different flow rates on cells	[153]
Shear stress	hK	High-throughput culture of HEK	[152]
Phalloidin, cytochalasin D, EGF	mFb	Assess wound healing	[96]
Electric stimulation, serum, β-lapachone	mFb	Assess wound healing	[172]
Shear stress, electric stimulation	hFb	Assess cell migration	[77]
N.A.	SB, HSE	Improve culture conditions for *in vitro* skin emulation	[147]
Doxorubicin, FAM-tagged nucleotide solution	HSE	Assess transdermal transport and skin toxicity	[146]
TNF-α for inflammation induction, dexamethasone	hK + hFb + HUVECs	Emulate skin oedema and drug testing	[148]
UV light, LPS, nickel sulfate, DNCB cobalt sulfate and glycerol	hK + human monocytic cell line	Assess the integrity of the skin barrier	[149]
Rat tail, porcine skin and duck feet collagen	hFb + hK	Assess the effects of different scaffolds on *in vitro* skin models	[179]
N.A.	hFb + hK + HUVECs	Improve culture conditions for *in* vitro skin emulation	[180]
SDS	RHE, mFb	Automate TEER measurements	[156]
Caffeine	hK + hFb	Assess transdermal transport	[163]
N.A.	hFb + hK	Improve culture conditions for *in* vitro skin emulation	[181]
Uniaxial stretching	hFb + hK	Develop skin ageing model	[169]
Fluorescein isothiocyanate-dextran and potassium dichromate	hK	Assess transdermal transport	[164]
Bacteria, penicillin	SB + human blood	Visualize neutrophil response to skin infection	[158]
Caffeine in a cream formulation	Mice and rat skin	Assess transdermal transport	[157]
Shear stress, β-lapachone	mFb	Assess wound healing	[155]
Sorafenib	hFb + hK	Assess drug toxicity	[165]
Topical application of 10 irritant and 10 non-irritant substances	hK + hFb + HUVECs	Assess skin irritation	[166]
Doxorubicin, dexamethasone, SDS, UV irradiation	hFb + hK + HUVECs + human leucocytes	Assess skin immune response (cytokine production and leukocyte infiltration)	[170]
Topical application of 10 chemicals	hK	Assess skin irritation	[150]
SDS	hFb + hK	Assess skin irritation	[167]
Electrical stimulation	mK	Assess wound healing	[151]
IL-4 and IL-3	hFb + hK	Develop atopic dermatitis model	[168]
Shear stress	mFb	Assess wound healing	[182]

*DNCB* dinitrochlorobenzene, *EGF* epidermal growth factor, *FAM* fluorescein amidite, *HEK* human embryonic kidney cells, *hFb* human fibroblasts, *hK* human keratinocytes, *HSE* human skin equivalent, *HUVECs* human umbilical-vein endothelial cells, *IL* interleukin, *LPS* lipopolysaccharide, mFb mouse fibroblasts, *mK* mouse keratinocytes, *N.A*. not applicable, *RHE* reconstructed human equivalent, *SB* skin biopsy, *SDS* sodium dodecyl sulfate, *TEER* transepithelial electrical resistance, *TNF* tumor necrosis factor, *UV* ultraviolet

The first microfluidic model with human keratinocytes was designed to provide a range of shear conditions to cells cultured over a collagen-patterned surface. Channels were designed with different lengths, resulting in a linear flow rate increase. This strategy allowed light microscopy analysis for cell morphology assessment, on-chip immunostaining and cell-viability quantification using fluorescence microscopy [152].

**Figure 3:**
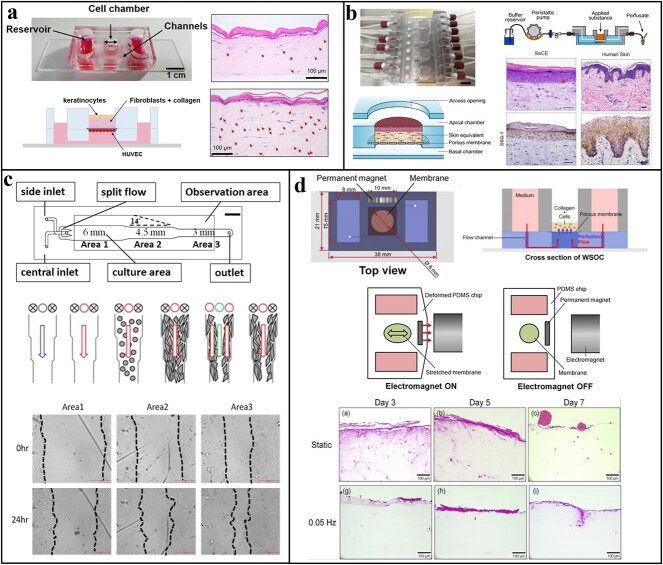
Design strategies for microfluidic skin-on-a-chip. (**a**) Schematic representation of the microfluidic skin chip (bottom left image); actual chip (top left image); and histological images of migration of leukocytes in response to UV irradiation (right images). Adapted from Kwak *et al*. [170]. Copyright (2020) Wiley. (**b**) Assembled organ-on-a-chip device in its bioreactor set-up (top left image); diagram of the full-thickness skin-on-a-chip model (bottom left image); diagram of the experimental setup (top right image); and histological images of the epidermal morphogenesis and expression of the DSG-1 protein (bottom right images). Adapted from Sriram *et al*. [163]. Copyright (2018) Elsevier. (**c**) Schematic representation of the microfluidic chip (top image); experimental set-up for wound creation using alternate flows of a washing buffer (blue) and trypsin (green) (middle images); and wound healing process of three wounds with different sizes under a flow of β-lapachone (bottom image). Adapted from Lin *et al*. [155]. Copyright (2019) Nature. (**d**) Illustration of wrinkled skin-on-a-chip (top left image); illustration of the perfusion of the chip (top right image); operation of the wrinkled chip (middle images); and histological images of the obtained skin models after static condition and stretching at 0.05 Hz (bottom images). Adapted from Lim *et al*. [169]. Copyright (2018) Elsevier *DSG-1* desmoglein-1*, HUVEC *human umbilical vein endothelial cells, *SoCE* skin-on-chip equivalents, *PDMS *polydimethylsiloxane, *WSOC* wrinkled skin-on-a-chip.

Recently, more sophisticated approaches have been developed, enabling, e.g. the inclusion of multiple skin layers in the same chip, as well as microvasculature. Kwak *et al.* developed a vascularized skin-on-a-chip comprised of dermis- and epidermis-like regions (Figure 3a) [170]. This chip was composed of two layers of PDMS separated by a membrane with a co-culture of fibroblasts, keratinocytes and endothelial cells. Through the use of a vascularized microfluidic system, it was possible to emulate cytokine production and sustain leukocyte infiltration [170].

Skin-on-a-chip research has also led to the development of improved models for wound healing, transdermal transport and skin irritation. Sriram *et al.* developed a full-thickness HSE microfluidic chip that enabled direct skin permeabilization and integrity assessment of multiple replicates (Figure 3b) [163]. This chip was composed of poly(methyl methacrylate) (PMMA) chambers for independent tissue culture and analysis. PMMA is mainly used in microfluidics due to its easy processability, cost-effectiveness, optical transparency and biocompatibility [171]. Each chamber had a permeable membrane separating the top and the bottom. For the construction of the skin-equivalent culture, dermal fibroblasts were first seeded in a non-contractible fibrin matrix on the porous membrane. Then, keratocytes were seeded on the top chamber with medium perfusion from the top and the bottom. Finally, perfusion only in the bottom of the chamber with ventilation on the top allowed the establishment of an air–liquid-interface culture [163]. Regarding wound healing assays, Sun *et al*. developed a PMMA chip for the assessment of the effect of chemical and electrical stimuli on fibroblast migration [172]. Briefly, this chip contained a top chamber for electric field generation and a bottom chamber for fibroblast seeding. The wound was performed using a barrier and healing was observed with time-lapse imaging [172]. Other microfluidic systems using the trypsin method to create a wound have also been developed. Recently, Lin *et al.* developed a sophisticated microfluidic system using this principle (Figure 3c) [155]. This chip enabled the creation of more than one wound per assay with different widths through different trypsin flow rates. This system allowed not only the evaluation of the effect of chemicals but also of physical stimuli for wound healing in a high-throughput manner [155]. For the assessment of skin irritation, Jeon *et al.* developed a system with three PDMS layers [166]. For validation of the model, 10 irritant and 10 non-irritant compounds were topically applied in the top layer. Afterwards, the predictive ability of irritant classification of this skin-on-a-chip was evaluated by analysing the tight junctions and cellular viability and compared with a RHE model. The results showed that this system has specificity and sensitivity in the range validated by the OECD [166].

The creation of skin-disease models is also of paramount importance to study disease onset and progression and for the evaluation of treatment options. Recently, Kim *et al.* emulated atopic dermatitis in a pumpless skin-on-a-chip [168]. This system was composed of a glass slide with two PDMS layers separated by a porous membrane. Although pumpless, the system allowed cell medium perfusion by gravity flow due to a rotating mechanism. After the co-culture of fibroblasts and keratocytes, the model was treated with inflammatory cytokines [168]. Moreover, Lim *et al.* created a wrinkled skin model that aimed to simulate aged skin using a magnet on the side of the cell chamber (Figure 3d) [169]. For wrinkle formation, an electromagnet was used to generate electromagnetic forces with the magnet, resulting in the uniaxial stretching of the cell culture. This microfluidic system may serve as a new model for future applications, including the testing of anti-wrinkling products by the cosmetic industry [169].

On-chip platforms for skin emulation have proven to be versatile alternatives for the evaluation of different biological phenomena. As they are more sophisticated than earlier *in vitro* models, they make it possible to study the combination of different stimuli and can be designed to be used with a wide range of skin models. Even though some miniaturization and process automation has been accomplished, to develop fully functional high-throughput on-chip systems, less manipulation and more integration of testing methodologies are required. Novel strategies combining high-throughput, organ-on-a-chip and advanced fabrication technologies, such as 3D bioprinting, have recently been reported [156,173–176]. However, testing reliability and reproducibility still have to be proven to possibly include on-chip assays in international testing guidelines. Recent developments in 3D bioprinting strategies and advanced bioinks will contribute to their integration with microfluidic devices towards the generation of sophisticated skin-on-a-chip models with superior clinical mimicry [177,178].

## Conclusions

The skin has a prominent role in maintaining the homeostasis of the human body and in protecting it from the outside environment. Due to its ease of access and complex barrier properties, a wide range of laboratory studies on skin are performed for research, product development and regulatory purposes. To achieve robust and comprehensive results, it is necessary to develop reliable testing platforms and methodologies. In recent years, there has been a shift away from *in vivo* animal studies towards faster, more affordable and human-relevant *in vitro* assays, with an increasing number and variety of them being included and recommended in internationally accepted testing guidelines. However, improvements are required and essential to make these assays more efficient and increase their predictive value. To that end, complementary approaches (e.g. high-throughput strategies, including microfluidic organs-on-a-chip), enabled by novel technological developments, have been increasingly adopted. For different types of studies, a balance between throughput, reliability and sophistication will need to be considered and achieved. This will be dependent on technological developments towards increasing complexity, automation and miniaturization.

## Funding

This work was supported by Portuguese funds from the Foundation for Science and Technology (FCT) (projects i3S, ref. UID/BIM/04293/2020; SKINCHIP, ref. PTDC/BBB-BIO/1889/2014; iMed.ULisboa, refs. UID/DTP/04138/2020 and UIDB/DTP/04138/2020); and UK funds from the Engineering and Physical Sciences Research Council (EPSRC) and Medical Research Council (MRC) (projects Centre for Doctoral Training in Regenerative Medicine, ref. EP/L014904/1, and Henry Royce Institute for Advanced Materials, refs. EP/R00661X/1, EP/P025021/1 and EP/P025498/1).

## Authors’ contributions

IVS co-wrote the manuscript, making a substantial, direct and intellectual contribution. MJSF conceived and contributed to the writing of the manuscript. LBB, SS and AFM reviewed the manuscript, making a direct and intellectual contribution to the work. RFP reviewed the manuscript, making a substantial, direct and intellectual contribution to the work (co-supervisor). PLG reviewed the manuscript, making a substantial, direct and intellectual contribution to the work, and approved it for publication (supervisor).

## Conflicts of interest

None declared.
